# Development of high-throughput ATR-FTIR technology for rapid triage of brain cancer

**DOI:** 10.1038/s41467-019-12527-5

**Published:** 2019-10-08

**Authors:** Holly J. Butler, Paul M. Brennan, James M. Cameron, Duncan Finlayson, Mark G. Hegarty, Michael D. Jenkinson, David S. Palmer, Benjamin R. Smith, Matthew J. Baker

**Affiliations:** 1 WestCHEM, Department of Pure and Applied Chemistry, University of Strathclyde, Technology and Innovation Centre, 99 George Street, Glasgow, G1 1RD UK; 2 ClinSpec Diagnostics Limited, University of Strathclyde, Technology and Innovation Centre, 99 George Street, Glasgow, G1 1RD UK; 3 Translational Neurosurgery, Department of Clinical Neurosciences, Western General Hospital, Edinburgh, EH4 2XU UK; 4 Institute of Translational Medicine, University of Liverpool & The Walton Centre NHS Foundation Trust, Lower Lane, Fazakerley, Liverpool, L9 7LJ UK

**Keywords:** Laboratory techniques and procedures, Translational research, Head and neck cancer

## Abstract

Non-specific symptoms, as well as the lack of a cost-effective test to triage patients in primary care, has resulted in increased time-to-diagnosis and a poor prognosis for brain cancer patients. A rapid, cost-effective, triage test could significantly improve this patient pathway. A blood test using attenuated total reflection (ATR)-Fourier transform infrared (FTIR) spectroscopy for the detection of brain cancer, alongside machine learning technology, is advancing towards clinical translation. However, whilst the methodology is simple and does not require extensive sample preparation, the throughput of such an approach is limited. Here we describe the development of instrumentation for the analysis of serum that is able to differentiate cancer and control patients at a sensitivity and specificity of 93.2% and 92.8%. Furthermore, preliminary data from the first prospective clinical validation study of its kind are presented, demonstrating how this innovative technology can triage patients and allow rapid access to imaging.

## Abstract (web summary)

Diagnosing brain cancer is frequently difficult and requires specialist equipment. Here, the authors develop their previous attenuated total reflectance-Fourier transform infrared spectroscopy method and incoporate the use of disposable silicon wafers for diagnosing brain cancer using serum samples.

## Introduction

The symptoms experienced by patients with a brain tumour are often non-specific and are more frequently associated with a benign non-tumour condition^1^. Headache, the most common symptom of brain tumours in adults^2^, also occurs in 4.4% of all primary care consultations^3^. Timely diagnosis of a brain tumour is therefore challenging. Brain tumours are the commonest cancer type to be diagnosed as an emergency presentation in the UK^1^, and many of these patients will have already seen their general practitioner (GP) several times.

It is difficult to determine which patients with non-specific symptoms are most at risk of having a brain tumour and should be prioritised for urgent medical imaging^4^. The best performing symptom-based referral guidelines for suspected brain tumour only expect to identify a brain tumour at a frequency of approximately 3% in the referral population. New strategies are needed to support decision making, particularly in primary care^5^.

**Fig. 1:**
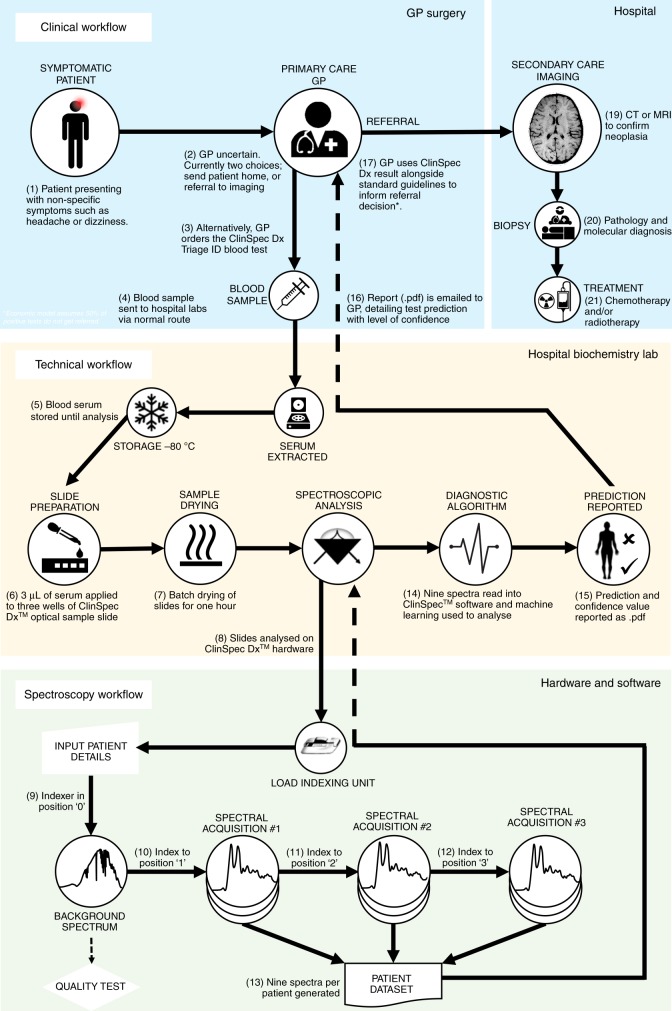
Proposed integration of a blood test for the triage of brain cancer. Lack of specific symptoms often results in brain cancer patients visiting their GPs numerous times before receiving a diagnosis; a blood test in primary care could effectively prioritise patients for urgent brain imaging, potentially reducing the time-to-diagnosis. The ATR-FTIR spectroscopy test would fit into standard blood analysis

A blood test at this point in the pathway could facilitate stratification of patients deemed at risk based on their symptoms, prioritising patients for urgent brain imaging (Fig. 1). It has been reported that the prevalence of brain tumours in this at risk population is as low as 1.6%, so a large proportion of scans are unnecessary^6,7^.

Attenuated total reflectance–Fourier transform infrared (ATR-FTIR) spectroscopy is a simple, label free, non-invasive, non-destructive analytical technique that can characterise the biochemical profile of a sample without extensive sample preparation. By interrogating biological samples with infrared (IR) light, it is possible to elucidate a specific biochemical fingerprint. ATR-FTIR spectroscopy has therefore demonstrable potential as a powerful diagnostic tool^8^. The interrogation of biofluids such as blood (and its derivatives, serum and plasma), can provide a systemic snapshot of the human body^9^ and reported diagnostic applications have included detection of malaria^10^, Alzheimer’s disease^11^, and a variety of cancers, including; ovarian^12^, colorectal^13^, lung^14^, and brain^15^.

In the brain tumour population, a comparison of blood serum has been made between patients with glioma and non-cancer patients^16^, between patients with high grade (glioblastoma) and low grade gliomas^17^, and in the largest study to date, samples from 433 patients explored a range of primary (glioma, meningioma) and secondary (metastatic) brain cancers^15^. These studies were performed on retrospective biobank collections of blood. The diagnostic output is generated by machine learning algorithms, which learn the differences in IR signatures that are indicative and exclusive to cancer. Much of the progression in this area is centred around these computational approaches which require fine tuning^18–20^. Based on these retrospective studies, the blood test yielded has a sensitivity and specificity for brain tumour of 92.8% and 91.5% respectively, using a traditional ATR-FTIR spectrometer configuration.

Conventionally, an ATR-FTIR spectrometer has a single point of analysis, the internal reflection element (IRE) is made from a material with a high refractive index, such as diamond, germanium, or zinc selenide. The serum sample, with a lower refractive index, is deposited directly onto the surface of the IRE^21^. IR light enters the IRE and is internally reflected, resulting in the formation of an evanescent wave at the IRE-sample interface. It is this wave that interrogates the samples at a defined penetration depth (calculable dependent upon refractive indices of the IRE and sample, the wavelength of IR beam, and the angle of incidence)^22^.

The primary limitation of the IRE is that they are usually high cost, inhibiting the production of an instrument with high-throughput capabilities. This has resulted in an inflexible technology, with a fixed point of analysis and no capacity for batch processing. The IR transparency and relative low-cost of engineering makes Silicon (Si) an ideal material for IREs^23^. For IR studies, Si IREs are not usually favoured due to Si lattice vibrations presenting within the biologically relevant fingerprint region, obscuring information below 1500 cm^−1^ wavenumbers^24^. These occur often due to larger sized crystals, as well as multi-bounce systems. Similarly to the phenomena of ATR-FTIR spectroscopy overcoming water absorption, by reducing the pathlength of the IR beam through the Si IRE, the contributions of the Si lattice vibrations can be minimised^25^. Microfabricated Si IREs may allow single bounce internal reflections, effectively minimising the IR beam pathlength and circumventing unwanted spectral contributions from Si.

The technical considerations inherent in applying this test in real-time in the clinic have been explored in great detail, including the examination of sample handling^26^, preparation^17,27,28^, and an investigation into the mechanisms of serum deposition patterns that may influence the spectral response^29^. The integration of the test into a clinical pathway has also been assessed^30^.

**Fig. 2:**
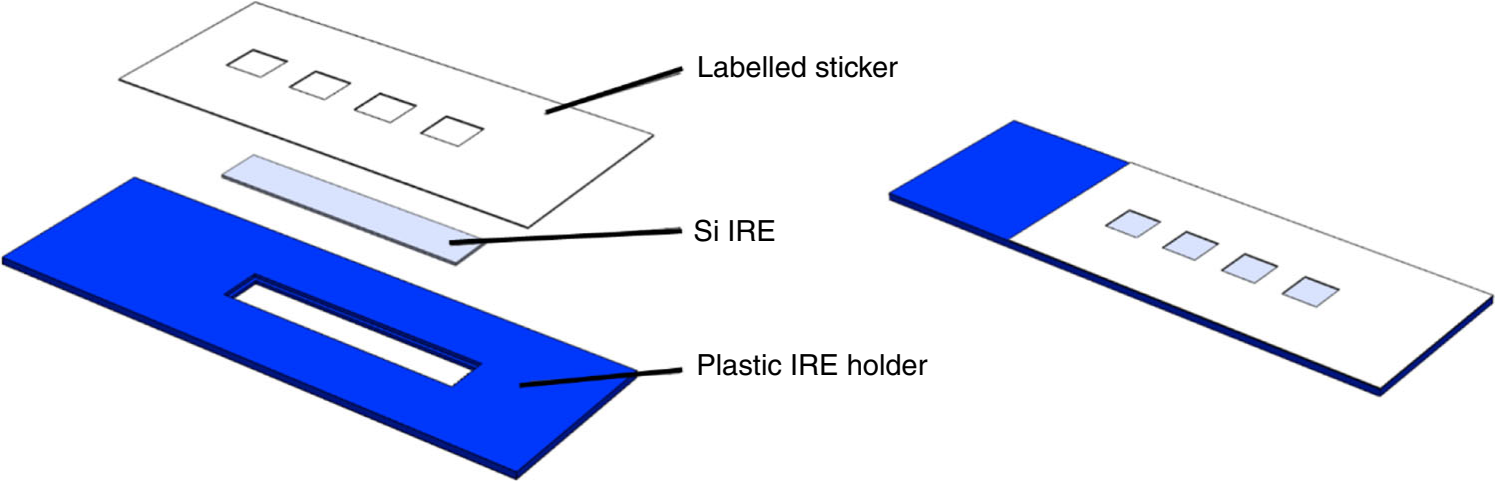
Expanded view of optical sample slides. Silicon internal reflection elements (Si IREs) are sandwiched between a 3D printed plastic holder and a medical grade labelled sticker. The latter defines the optically active regions of the Si IRE, split into four wells, three used for triplicate sample analysis and one for a background measurement. The fully assembled slide measures 75 mm × 25 mm, mimicking standard microscope slides

In our proposed pathway for patients with suspected brain tumours (Fig. 1) the result derived by ATR-FTIR spectroscopy is not used as an absolute diagnostic, rather a triage tool, which provides the GP with additional information to inform their referral decision^31^. We develop disposable Si IRE sample slides that allow the rapid preparation and analysis of multiple samples, enabling high-throughput ATR-FTIR spectroscopy optimised for clinical research (Fig. 2). These can be purchased at the fraction of a cost of a traditional IRE. Based upon the design of a microscope slide, these optical sample slides contain a Si IRE with four sample areas; one for background measurements and three for repeat measurements of a single patient. This device is developed for the triplicate measurement of patient samples with optimised spectral throughput and performance. We describe the transition to this technology for the established application of ATR-FTIR spectroscopy of blood serum for the detection of brain cancer, and the subsequent impact on clinical translation. This technological advance has led to a prospective clinical validation study in the target population, the interim analysis is presented here. The translation of this blood test for the early detection of brain cancer into to the clinic depicts both a technological step and a step towards to improving the clinical pathway for patients with this disease.

## Results

### Si IRE evaluation

**Fig. 3:**
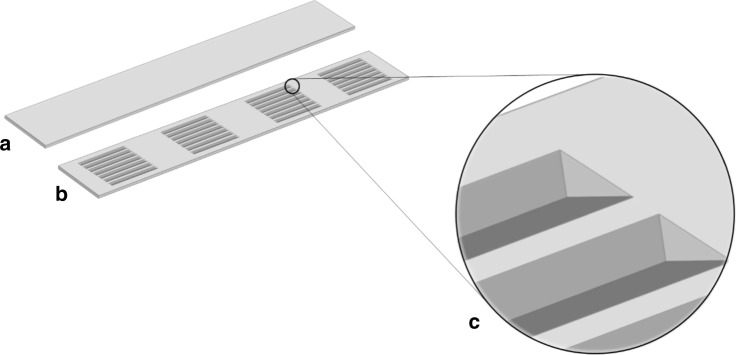
Schematic representation of Si IREs for clinical spectroscopy. **a** Sample-side, **b** IR facing side, **c** v-groove detailing (not to scale)

Defined arrays of v-grooves were successfully microfabricated onto Si wafers. These arrays define the optically active region of the Si IRE, where serum is deposited and subsequently analysed using IR light. The etched surface of the Si IRE is IR beam facing, so that light is coupled through the Si IRE parallel to the direction of the grooves (Fig. 3).

The etching process is highly accurate, with etching efficiencies ensuring that optical features of the Si IREs are accurate within error margins below 2%. This minimises the risk of increased spectral variability that may arise due to inconsistencies in the optically active regions of the disposable IRE. An in-house derived protocol has been developed to quality test freshly produced Si IREs, ensuring that the background energy spectrum falls within a defined acceptance range.

**Fig. 4:**
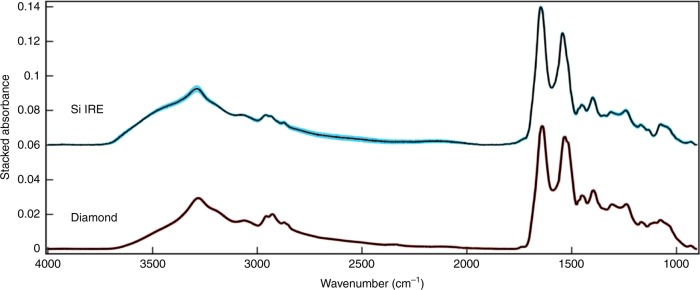
Human pooled serum spectrum obtained from Si IRE ATR-FTIR spectroscopy. Coloured shading represents standard deviation of ten spectra derived from Si (blue) and diamond (red) IREs

To explore the analysis of human blood serum, the spectral output of Si IREs were compared against conventional ATR-FTIR spectroscopy using a diamond IRE (Fig. 4). In this example, human pooled serum was analysed to remove biological variation. Spectra derived from Si IREs display increased variation between spectra, shown by larger standard deviation shading surrounding the mean spectrum. This is predominantly visible between 2700 and 2000 cm^−1^ wavenumbers, although few biological peaks are present in this region of the spectrum. The fingerprint region (1800–1000 cm^−1^) is considered the region of biological interest and marginal increases in variation are visible, although not significantly more than diamond obtained spectra. Some spectral features differ slightly between the two IREs; noticeably, the shoulder of N–H protein band (3500–3300 cm^−1^), the ratio between the amide I and amide II peaks, and contributions from the Si lattice vibrations (1100 cm^−1^).

### Retrospective patient cohort

A 724 retrospective patient cohort was investigated to assess the diagnostic performance of Si IRE based ATR-FTIR spectroscopy. This cohort contains a range of primary and secondary brain cancers as well as control (non-cancer) patients (Table 1). Glioblastoma multiforme (GBM) is the largest primary cancer group in this study, reflecting the higher incidence of this tumour class. Where possible patients have been age and sex matched between both disease groups, whilst also taking into account the natural disease prevalence (Table 2)^32^. An age-matched subset of this cohort (*n* = 226) was also analysed in order to ensure that any differentiation between disease groups is associated with cancer presence only (Supplementary Table 1 and Supplementary Fig. 3).

**Table 1:** Retrospective patient cohort breakdown with tumour classification details

WHO classification	Tumour type	WHO grade	Total
Tumour			
Diffuse astrocytic and oligodendroglial tumours	Glioblastoma multiforme	IV	260
	Gliosarcoma	IV	4
	Oligodendroglioma	II	11
	Diffuse astrocytoma	II	23
	Anaplastic astrocytoma	III	10
	Oligoastrocytoma	II	3
	Glioma	I	7
Other astrocytic tumours	Pilocytic astrocytoma	I	9
	Pleomorphic xanthoastrocytoma	II	1
Tumours of the cranial and paraspinal nerves	Schwannoma	I	14
Ependymal tumours	Ependymoma	II	6
Mesenchymal, non-meningothelial tumours	Haemangiopericytoma	II/III	2
	Haemanglioblastoma	I	1
Neuronal and mixed neuronal-glial tumours	Ganglioglioma	I	1
Embryonal tumours	Medulloblastoma	IV	1
Tumours of the pineal region	PPTID	II/III	1
Meningiomas	Meningioma	I	46
Pituitary tumours	Pituitary adenoma		29
Lymphomas	Lymphoma		2
Metastatic tumours	Metastasis		56
Control			237
		Total	724

**Table 2:** Retrospective patient cohort information

	Cancer	Non-cancer
Total	487	237
Sex (M/F)	280/207	149/84
Age range	21–96	19–69
Average age	61	35

**Fig. 5:**
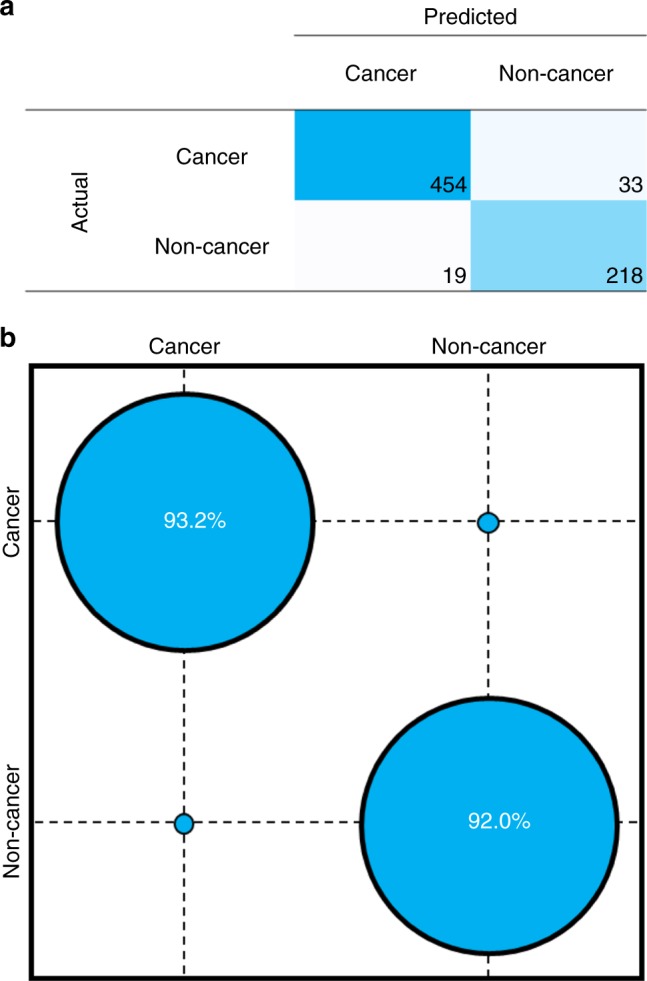
Disease prediction of blood serum using ATR-FTIR spectroscopy coupled with SVM classification. **a** Confusion matrix of classification on a per patient basis with **b** confusion ball visualisation of sensitivity and specificity. All data refer to resampled and averaged test set predictions

Using machine learning, it is possible to predict disease status from spectral data by training a model on a known dataset (known as supervised learning), and subsequently testing on a blind test dataset. The prediction results from a support vector machine (SVM) classification are presented here, and are indicative of the potential predictive power of ATR-FTIR spectroscopy coupled with machine learning. Binary classification between cancer and non-cancer is explored primarily to portray the suggested use as a triage tool. A prediction is made for each of the nine spectra from a single patient and the majority vote is taken as the diagnostic prediction for that patient (cancer or non-cancer). The accuracy of the classifier can be deduced by observing the number of correct and incorrect predictions for an external test set containing data that was not used in training the algorithm (Fig. 5). On a per patient basis, and averaged over 51 randomised training and test set splits, the sensitivity and specificity of the approach computed for the test set is 93.2% and 92.0% respectively, indicating potential to detect both cancer and control patients effectively. When considering an age-matched subset of this cohort, an equally high sensitivity and specificity of 89.5% for both metrics indicates this promising performance is likely associated with disease differentiation specifically, as opposed to a difference in cohort age or sex. The area under the curve (AUC) of this model, as derived from the receiver operating characteristic (ROC) curve (Supplementary Fig. 2) has a value of 0.96, which can be considered excellent.

### Prospective clinical validation study

With promising results obtained from a retrospective patient dataset, the suitability of the test in a clinical setting was assessed in a prospectively recruited patient cohort. Patients referred from their GP for brain imaging to exclude a brain tumour were recruited, along with patients with a recent diagnosis of a new brain tumour admitted to the neurosurgical unit. The outputs from this study were two-fold; firstly, a blinded analysis of the trained patient algorithm and secondly, a better understanding of unknown patient variance that can arise from prospective cohorts.

**Fig. 6:**
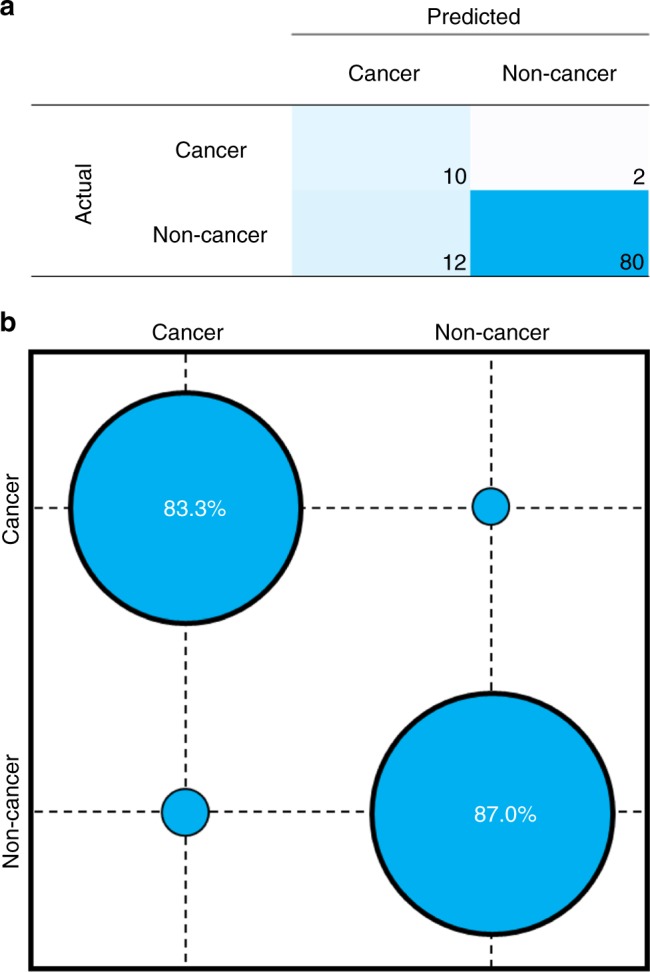
Disease prediction of a prospective patient cohort with suspected brain cancer using ATR-FTIR spectroscopy. Blind analysis was conducted using an SVM algorithm previously trained on retrospective patient cohort. **a** Confusion matrix of classification on a per patient basis with **b** confusion ball visualisation of sensitivity and specificity

For this process the previously described SVM algorithm, trained on the 724 retrospective cohort, was used to predict the disease status of 104 test patients recruited as part of prospective clinical validation study (Fig. 6), in the first interim analysis of the cohort. Throughout this process, the analysis was blinded to the imaging outcome until the algorithm result had been reported. The reported sensitivity of 83.3% and specificity of 87.0% display noticeable reductions when compared to retrospective patient results. This initially would suggest diminished ability to detect patients with or without cancer; however, the value for sensitivity falls above the thresholds for cost-effectiveness already defined^33^. The same can also be said for specificity, where a value above 80% indicates the test would provide cost savings to the UK and US health services when used as a triage tool in secondary, as well as primary care.

Within this prospective patient cohort, a total of 12 patients were observed to have cancer. The tumour type breakdown included; four GBM, three anaplastic astrocytoma, two oligoastrocytoma, one medulloblastoma, one ependymoma and one gliosarcoma. It is clear in Table 1 that some of these tumour classifications are not well represented in the training dataset and could thus be the source of unwanted false negative predictions. For example, the ependymoma case was incorrectly identified as non-cancer (data not shown) and only six incidences of this tumour class are found in the 724 patient training dataset.

## Discussion

A simple test for the early diagnosis of brain cancer in primary care has the potential to make dramatic impacts on patient survival and quality of life, whilst also saving resources and costs within health services across the world^15^. Analysis of blood serum using ATR-FTIR spectroscopy is a technique advancing towards the clinic, aiming to fill the void as a triage tool to help inform GP referral decisions^33^. Up until now, the technique has been limited by low-throughput technology that requires near constant user attention, potentially inhibiting clinical uptake. Disposable, Si IREs transform conventional ATR-FTIR spectroscopy by allowing batch processing of samples, and conversion of commercial spectrometers into high-throughput analysers.

Integration of Si IREs into spectrometers is simplified by patent pending technology; optimised design of the Si IRE holder allows easy handling, and an indexing unit guides the slide across the open aperture of the spectrometer. The spectral output of Si IREs is of equivalent quality to that of convention IRE materials, such as diamond, without exhibiting strong spectral features from the Si itself. Whilst there is increased variability between spectra obtained with Si IREs, this is minimal for an approach where the background reference spectrum is taken from essentially a separate IRE. Differences between diamond and Si IREs can be attributed to differing baselines as a consequence of the grooved under surface; however, this can be modelled using iterative processes.

The classification performance of the Si IREs on a retrospective dataset is also equivalent to previously reported data on diamond. The 724 patient cohort analysed using Si IREs obtained a sensitivity and specificity of 93.2% and 92.8% respectively, compared to 92.8 and 91.5% on a retrospective 433 patient cohort^18^. In both these examples, a SVM-based classification protocol was used to extract the signals of primary and secondary brain cancers. Although the patient cohorts were comprised of separate patients and thus not entirely comparable, the high diagnostic capability is positive. Improved sensitivity of the technique may occur as a consequence of altering the IRE material and thus the penetration depth of the evanescent wave, or due to accounting for more variance in the patient population.

With confirmation of diagnostic performance on Si IREs, the retrospective dataset is fundamental to predictions of prospective patients. By training a classification algorithm on this known population, blind data can be predicted based upon the derivation of cancer signals from the dataset. This approach would mimic the real-world application of the blood test and is thus an appropriate measure of true diagnostic accuracy. The first prospective study employing ATR-FTIR spectroscopy is presented here; an interim analysis of an ongoing prospective validation study.

A sensitivity of 83.3% shows the huge clinical potential of this test, effectively identifying those patients who desperately need brain scans. From a patient perspective, higher sensitivity has the greatest impact on quality of life due to the opportunity to identify and treat cancers early^34^. From an economic perspective, a test with high specificity is desirable as the number of unnecessary brain scans can be reduced; estimated to be in the region of £6–12 million every year in the UK alone^33^. At a specificity of 87.0%, the introduction of an ATR-FTIR spectroscopy based blood test in primary care would deliver significant cost savings to the health services.

It is important to note, that this preliminary clinical data provides an early insight into the diagnostic accuracy of this blood test, with the caveats of small patient numbers (*n* *=* 104) which limits the obtainable values of sensitivity and specificity. Considering the data with regards to the potential reduction in unnecessary brain scans, in this cohort of 104 patients, only 12 patients would be referred to secondary care. Taking into account the health economic argument that 50% of patient would still be referred by their GP, regardless of the spectroscopic blood test result, this would still result in a 44% reduction in the number of brain scans^33^.

This work presents a step in the translation of ATR-FTIR spectroscopy into the clinic. This step towards high-throughput analysis has implications in the field of IR spectroscopy as well as the clinical environment. Analysis of blood serum using this technique would fit ideally in the clinical pathway as a triage tool for brain cancer. For the effective treatment of this disease, it is essential to identify tumours early—A need presently unmet by the current diagnostic pathway. A triage tool available in primary care would provide GPs with further information to inform their referral decision; providing at risk patients access to imaging in secondary care, whilst also providing reassurance to those patients who are not at risk. Consequently, time-to-diagnosis for brain cancer patients could be reduced whilst also bringing cost benefits to the health services.

## Methods

For thorough understanding of this issue, we direct the reader to key literature regarding instrumentation and theory of ATR-FTIR spectroscopy^8,22,35^.

### Si IRE production

Si IREs were designed in-house, and optimised for spectral collection in the mid-IR wavelength region between 2.5 and 25 µm. Dimensions of the Si IREs were chosen based on optimum light throughput, spectral quality and reproducibility. An array of v-grooves are etched into the IR facing surface using conventional anisotropic wet etching following the stages of photomask design, hard mask deposition, application of photoresist and hard mask etching. Upon etching, Si wafers are diced into individual four-well Si IREs. These Si IREs are placed into plastic IRE holders, developed and 3D printed in-house, and held in place with an adhesive top layer, that also defines the deposition areas on the sample surface (Fig. 2). Once assembled, this constitutes the optical sample slide.

A custom built translational stage was constructed that interfaces optical sample slides with commercially available FTIR spectrometers. These stages can be automated to further improve throughput, reducing the personnel resource required.

### Retrospective patient cohort

To compare the spectral, and thus diagnostic, capabilities of Si IREs with traditional IREs a large cohort retrospective study was conducted on control and cancer patients. Samples were obtained from three sources; the Walton Centre NHS Trust (Liverpool), Royal Preston Hospital (Preston), and also from the commercial source Tissue Solutions Ltd (Glasgow, UK). Ethical approval for this study was obtained (Walton Research Bank BTNW/WRTB 13_01/ BTNW Application #1108) and informed consent was obtained from all patients prior to serum collection. Inclusion criteria for cancer patients were as follows: (1) patients with pathologically confirmed primary or secondary brain cancers, (2) no chemo- or radio-therapy at the time of tissue sampling. For control patients, inclusion criteria stated that the person should have no history of cancer, or be undergoing any medical treatments. A total of 724 patients were included in this study. Blood samples were obtained in S-Monovette (Sarstedt, Germany) collection tubes and allowed to clot for up to one hour, before being centrifuged at 2200 × *g* for 15 min at room temperature. Serum was subsequently aliquoted and frozen at −80 °C until the time of analysis.

### Prospective clinical study

To compare the diagnostic accuracy of the blood test, including hardware and software, a prospective validation study was established at the Western General Hospital, Edinburgh, with local ethical approval (2017/0320/SR938, 15/ES/0094). Informed consent was obtained from all patients prior to serum collection. Lothian region patients were eligible if suspected of having a brain tumour by their GP and referred directly for brain imaging through the local open access CT (OACT) service. This is most closely representative of the target patient cohort for our triage blood test. The criteria for GPs to access the OACT pathway is simply that their assessment that a patient’s symptoms necessitate urgent brain imaging; patients requiring emergency imaging are referred to the Emergency Department or indeed via a stroke referral pathway. As such these patients represent a cohort of patients the GP has selected for referral brain imaging. Patients referred via the OACT route for brain imaging were eligible to take part in this validation study provided they were able to give informed consent. Patients with a new brain tumour diagnosis referred to the neurosurgical unit for assessment were also eligible for inclusion. Analysis of the blood samples with our strategy was blinded to the whether the patient had a brain tumour or not. Sample handling protocols were consistent with those from the retrospective patient cohort previously mentioned.

The study was developed to have an adaptive design, to ensure that the optimum number of patients was recruited to enable assessment of test sensitivity and specificity. For initial sample size estimates (80% power, 5% significance level), to estimate specificity within 5% required a sample size between 200 and 600 assuming 2% prevalence (or 98% without brain tumour) for an assumed specificity in the range of 95% (*n* = 188) to 75% (*n* = 622). For the sensitivity, to demonstrate 90% sensitivity to within 30% requires 400 subjects (3% prevalence) or 600 (2% prevalence); note specificity was 91% in our previous study. The sample size was planned to be calibrated throughout the study by scheduled interim sample analysis and the cohort presented here is the first patient group from the larger study to be analysed. With a prevalence of 1–3% of actual brain tumours amongst imaged patients, we expected to generate evidence on specificity (true negatives and false positives) far more quickly than for sensitivity (true positives and false negatives). An estimate of the overall specificity of the blood test was made by correlating the predicted diagnosis with the gold standard (brain imaging, and biopsy where necessary). This interim analysis was made within the adaptive element of the design to assess how many additional samples would be needed in the full study to estimate specificity. Supplementary Fig. 1 shows a consort flow diagram of the validation study and Supplementary Table 2 shows the demographic data for patients that either accepter or declined to be part of the study to highlight the unbiased approached to patient recruitment.

### Sample preparation

Serum samples were thawed at room temperature (18–25 °C) for a minimum of 15 min. Serum was deposited onto the three sample wells of the optical sample slide, ensuring that the background well remains empty. Prepared slides are placed in a drying unit (Thermo Fisher Heratherm, GE) Incubator at 35 °C for 1 h, providing even heat and airflow for controlled drying dynamics of the serum droplet^29^.

### Spectral collection

The optical sample slides act as both a sample substrate and a versatile IRE for IR spectroscopy, and can be easily interfaced with commercial spectrometers. For this study, a specular reflectance accessory combined with the optical sample slides replaces traditional IREs. A purpose built ‘Slide Indexing Unit’—a simple slide carriage connected to a linear motor—allows accurate movement across the spectrometer aperture, indexing the optical sample slide between wells. This automated unit can ultimately provide high-throughput analysis, that does not require constant attention from technical staff. Spectra were collected using optimum spectrometer parameters for Si IREs, defined in this instance as 4 cm^−1^ resolution (with 1 cm^−1^ data spacing) and 16 co-added scans.

### Spectral analysis

Data handling is fundamental to this technology, and forms a critical part of intellectual property and know-how regarding Si IREs for clinical use. In short, spectral analysis can be divided into spectral pre-processing and spectral classification; the former reducing unwanted variance in the dataset and the latter performing the disease prediction step.

For pre-processing, a combination of baseline correction, normalisation and data reduction enables the significant biological information, in this case disease status, to be revealed and improves the classification performance^20^. The optimum pre-processing protocol was determined using a trial-and-error iterative approach and is specific to Si IRE analysis.

For the spectral classification, machine learning techniques were applied to the spectral datasets. The purpose of this approach is to identify the signals of cancer from a known patient cohort to develop a trained classification model, and then to use this information to predict the presence of cancer in an unknown population; as per the real-world application. In this instance, the retrospective patient cohort would form the ‘Training’ dataset and the prospective patient cohort would form the external ‘Testing’ dataset. In this study we present the use of a SVM learning to extract spectral patterns indicative of cancer. SVMs are supervised techniques that construct a hyperplane through data projected in a high dimensional space, allowing classification of spectra and or patient data^36^.

To train the classification models, patients from the retrospective patient cohort were randomly split into training (70%) and test (30%) portions. No single patient can appear in both of these portions. Algorithmic parameters were tuned using a grid search to maximise Kohen’s Kappa computed on a per-spectra basis for five fold cross validation on the training set. The tuned model was then used to make predictions for the spectra in the external test set. Each of the nine spectra generated per patient was analysed independently, with the consensus vote amongst the spectra reported as the diagnostic outcome (cancer or non-cancer). The whole process was iterated 51 times to obtain adequate sampling for summative statistics. The performance metrics, such as sensitivity and specificity, that are reported in the Results section were derived based on the number of correct and incorrect predictions for the external test set only.

To then predict the prospective patient cohort, the iterated machine learning algorithm(s) were again trained on the training portion of the 724 retrospective patient cohort, internally validated using the test set, and subsequently applied to a blind external test set; in this case, the prospective patient cohort. The diagnostic outcome for each patient was reported as the consensus vote of all predictions.

### Reporting summary

Further information on research design is available in the Nature Research Reporting Summary linked to this article.

## Supplementary information

Supplementary Information

Reporting Summary

## Data Availability

The datasets generated and/or analysed during the current study are available within the article and Supplementary Information files and available from the authors upon reasonable request.
