# Supplementary material for: Development of high-throughput ATR-FTIR technology for rapid triage of brain cancer
Source: Nat Commun. 2019 Oct 8;10:4501. doi: 10.1038/s41467-019-12527-5 (PMC6783469; doi:10.1038/s41467-019-12527-5)
Supplement: Supplementary file 1 — Supplementary Information [file 41467_2019_12527_MOESM1_ESM.pdf]

Supplementary Information

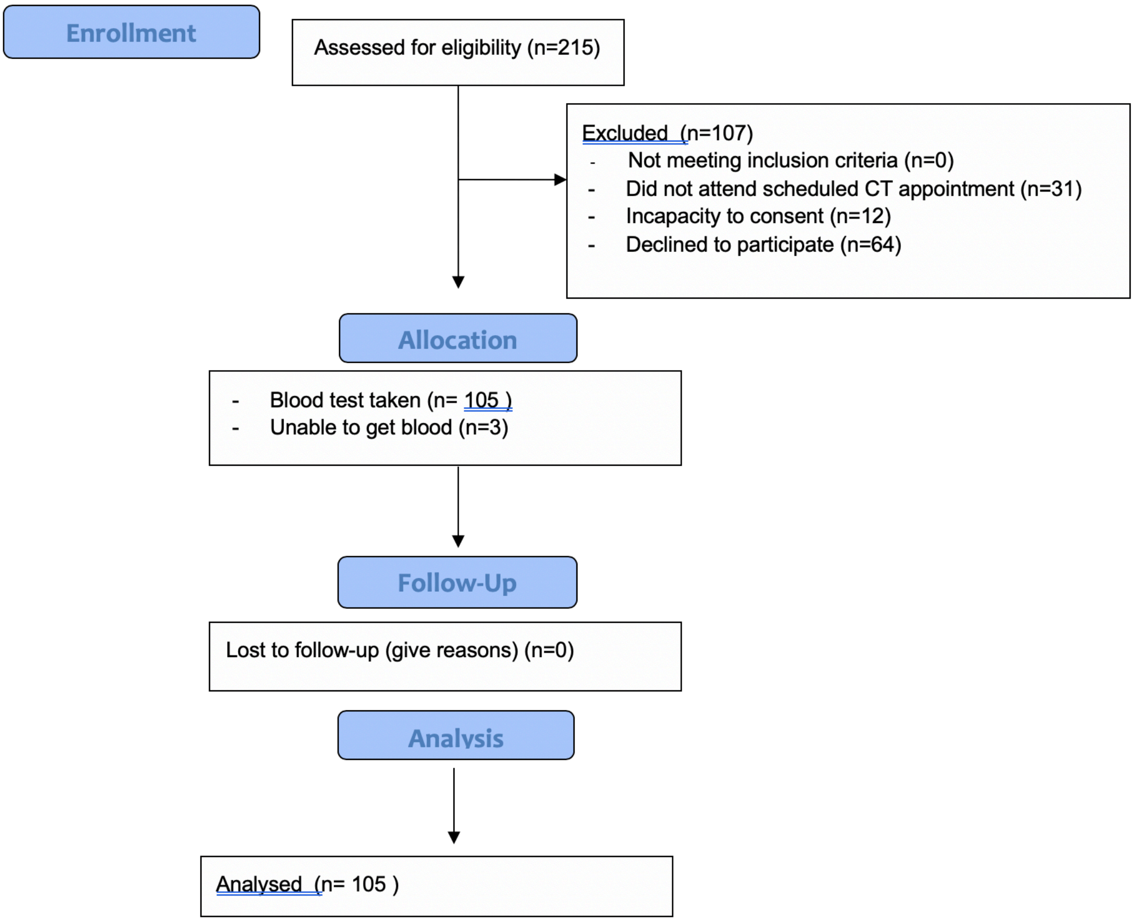

Supplementary Figure 1. CONSORT Flow Diagram for validation study.

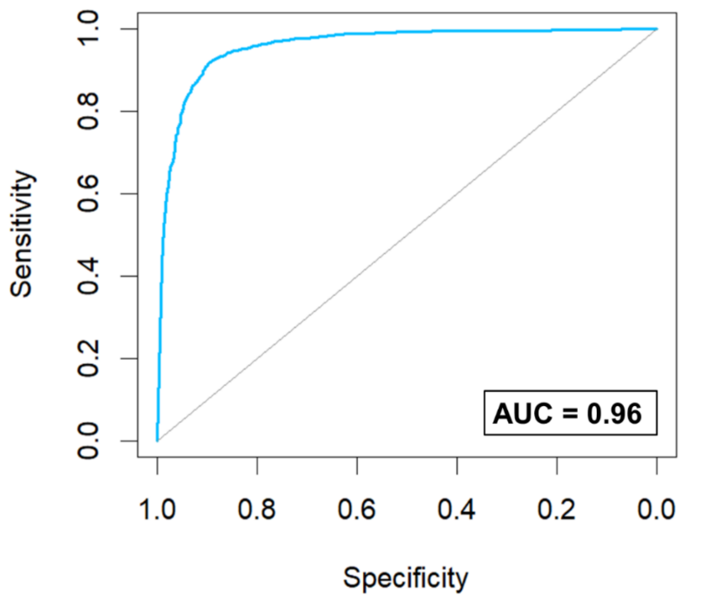

Supplementary Figure 2. Receiver operating characteristic (ROC)-like curve displaying trade-off between sensitivity and specificity of SVM classification of spectra derived from brain cancer and non-cancer patients.

Receiver operating characteristic (ROC) curves can discern the discriminatory ability of a machine learning approach by presenting the interplay between false positive and true positive rates. ROC curves, observing sensitivity and specificity, for SVM classification of the retrospective patient dataset indicate that diagnostic performance of the blood test alongside machine learning is promising (**Supplementary Figure 2**.). There is little trade-off between sensitivity and specificity, shown by the symmetrical form of the curve with only a slight skew towards better sensitivity. This may occur as a consequence of patient split between non-cancer and cancer; however, spectral processing has minimised the impact of this by accounting for any data imbalance. The area under the curve (AUC) also depicts the high diagnostic capabilities of the test, with a value of 0.96 considered excellent.

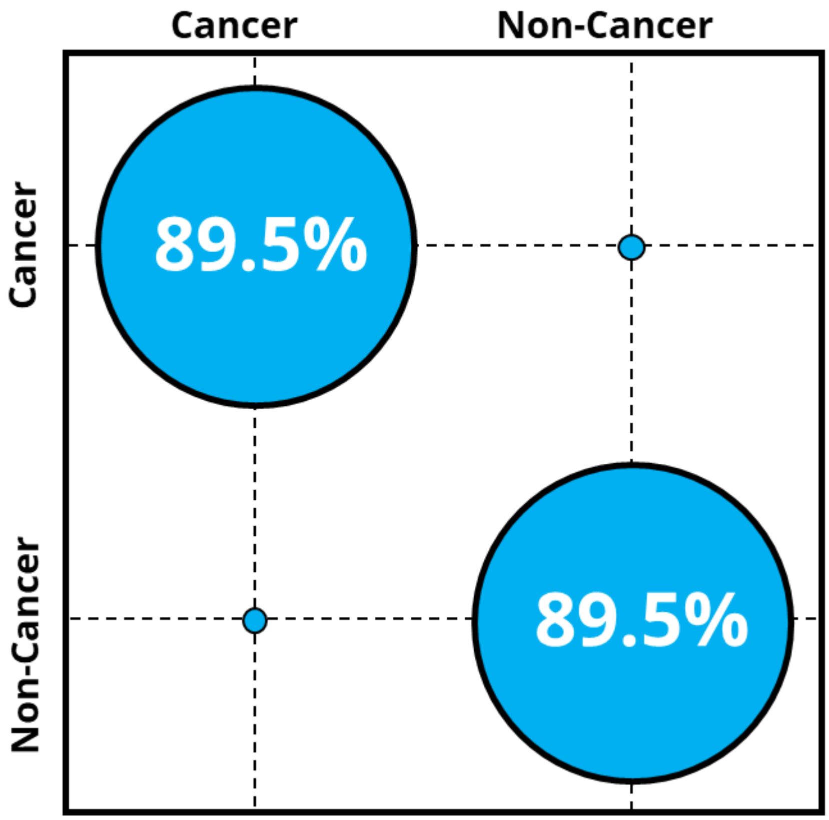

Supplementary Figure 3. Confusion matrix of external test set predictions of an age matched subset of the 724 patient retrospective cohort.

Supplementary Table 1. Retrospective, age and sex matched patient cohort information

	Cancer	Non-Cancer
Total	128	128
Sex (M/F)	78/50	78/50
Age Range	19-69	19-69
Average Age	42	42

Supplementary Table 2. Demographic data for patients who were either declined or recruited into the study

	Age	N Male	N Female
Declined	63	37	71
Recruited	58	42	57
